# Modeling the probability distribution of positional errors incurred by residential address geocoding

**DOI:** 10.1186/1476-072X-6-1

**Published:** 2007-01-10

**Authors:** Dale L Zimmerman, Xiangming Fang, Soumya Mazumdar, Gerard Rushton

**Affiliations:** 1 Department of Statistics and Actuarial Science and Department of Biostatistics and Center for Health Policy and Research, University of Iowa, Iowa City, IA 52242, USA; 2 Department of Statistics and Actuarial Science, University of Iowa, Iowa City, IA 52242, USA; 3 Department of Geography, University of Iowa, Iowa City, IA 52242, USA

## Abstract

**Background:**

The assignment of a point-level geocode to subjects' residences is an important data assimilation component of many geographic public health studies. Often, these assignments are made by a method known as automated geocoding, which attempts to match each subject's address to an address-ranged street segment georeferenced within a streetline database and then interpolate the position of the address along that segment. Unfortunately, this process results in positional errors. Our study sought to model the probability distribution of positional errors associated with automated geocoding and E911 geocoding.

**Results:**

Positional errors were determined for 1423 rural addresses in Carroll County, Iowa as the vector difference between each 100%-matched automated geocode and its true location as determined by orthophoto and parcel information. Errors were also determined for 1449 60%-matched geocodes and 2354 E911 geocodes. Huge (> 15 km) outliers occurred among the 60%-matched geocoding errors; outliers occurred for the other two types of geocoding errors also but were much smaller. E911 geocoding was more accurate (median error length = 44 m) than 100%-matched automated geocoding (median error length = 168 m). The empirical distributions of positional errors associated with 100%-matched automated geocoding and E911 geocoding exhibited a distinctive Greek-cross shape and had many other interesting features that were not capable of being fitted adequately by a single bivariate normal or t distribution. However, mixtures of t distributions with two or three components fit the errors very well.

**Conclusion:**

Mixtures of bivariate t distributions with few components appear to be flexible enough to fit many positional error datasets associated with geocoding, yet parsimonious enough to be feasible for nascent applications of measurement-error methodology to spatial epidemiology.

## Background

It is becoming increasingly common in public health studies to use the spatial locations of study participants in statistical analyses, for example to test for geographic clustering of disease or to estimate relationships between environmental exposures and disease. Indeed, statistical methods for spatial epidemiology are developing rapidly, and the growing list of book-length treatments of the subject include [1-4]. In order to utilize subjects' locations in a spatial analysis, it is necessary, of course, to define and ascertain these locations. Historically, the spatial location of a person has been defined as the person's place of residence; however, recognition of human mobility and the fact that many causative exposures occur outside the home have generated recent attempts to expand this definition to daily activity spaces and such constructs as time geography and pathogenic paths; for a brief review see [5]. Nevertheless, place of residence currently remains the typical representation of each subject's location in public health studies.

The spatial coordinates of a place of residence are usually not measured directly; rather, the residential address is given a location reference, known as a geocode. The geocode may be defined as the latitude and longitude coordinates or a point in some other coordinate system, or as a statistical tabulation area such as a U.S. Census tract, block group, or block. Here, unless noted otherwise, we use the point rather than areal definition. Several distinct methods for geocoding exist, including visiting the residence with global positioning system (GPS) receivers, identifying the residence on orthophoto maps based on aerial imagery, and matching the address to a digital street map. The latter can be done in batch mode for large numbers of addresses and when done this way is often called "automated geocoding." Recently, a new method of automated geocoding has been developed that matches an address to parcel descriptions of legal property boundaries developed by assessors, but this method has not yet been widely adopted. The U.S. Census Bureau is developing such a parcel-level geocode for all U.S. addresses, but the public does not and will not have access to these geocodes. Accordingly, automated geocoding here will refer to the widely used practice of using a geographic information system (GIS) to match an address to a street name and address range in a digitized street reference map and then estimate, via interpolation, where the address is located between the two points that define the limits of the address range.

Automated geocoding is cheaper, more convenient, and hence much more common than non-automated methods, but considerably less accurate. Several investigations of the accuracy of automated geocoding have recently been published. Some of these have measured accuracy by the proportion of addresses for which the geocode belongs to a correct statistical tabulation area; for example, Yang et al. [6] and Kravets and Hadden [7] found that only 70% to 90% of their geocoded addresses were assigned to the correct census block. Other investigations have measured accuracy by the Euclidean distance between the point location ascertained by automated geocoding and the corresponding "true" location as determined by a much more intensive and accurate method (e.g. GPS receivers or aerial imagery) [8-13]. These latter studies have shown that positional errors of several hundred meters are incurred regularly by automated geocoding, and that even larger errors are not uncommon in rural areas. In one of the most thorough studies of automated geocoding errors published to date, Cayo and Talbot [14] found that 10% of a sample of rural addresses in a four-county upstate New York study area geocoded with errors of more than 1.5 km, and 5% geocoded with errors exceeding 2.8 km.

An alternative method of geocoding that may have promise for public health research is E911 geocoding. E911 geocodes are usually obtained under the auspices of local governments for the specific purpose of dispatching emergency vehicles to the correct location in response to a 9-1-1 telephone call requesting assistance. The particular methods used to obtain the geocodes vary, but they generally are more resource-intensive than mere automated geocoding due to the life-and-death issues at stake. For example, some counties have used parcel address-matching, while others have hired commercial firms that claim to take a GPS measurement at or near each residence. Every year, more counties in the U.S. develop E911 geocodes, so it is possible that in the not-too-distant future, many health researchers will be able to use these geocodes in lieu of performing automated geocoding. Investigations of the accuracy of E911 geocodes have not yet appeared in the scientific literature, though commercial firms offering E911 geocoding services tout them, unsurprisingly, as much more accurate than geocodes obtained via automated geocoding.

Whatever process is used to obtain geocodes of residences, the positional errors incurred by that process introduce location uncertainties that may adversely affect spatial analytic methods. Specific effects of positional errors on spatial statistical analyses include inflation of standard errors of parameter estimates and a reduction in power to detect such spatial features as clusters and trends [15-17]. Even relatively small positional errors can have a discernible impact on local statistics for detecting clustering or "hot spots" [18]. It is important, therefore, for researchers to quantify these effects on their analyses, which in turn requires them to have, or gain, some understanding of the probability distribution of the positional errors. In fact, the adoption of an adequate model for the distribution of positional errors is essential for successful implementation of existing measurement-error model methods for spatial data analysis; see, e.g., [19-22]. Knowledge of the error distribution also facilitates the use of multiple imputation methods for adjusting spatial statistical analyses for positional errors. These methods proceed by imputing (simulating) locations with error from the distribution of an observed location given its corresponding true location. Inferences for the spatially-varying health outcome of interest can then be made using the model for that outcome given the true locations, but with each true location replaced by multiple imputed realizations. Finally, gaining an understanding of typical geocoding error distributions allows for the simulation of realistic positional errors for power studies of various tests for clusters, spatial trends, and other important spatial patterns and features.

The main purpose of this article is to formulate and fit useful models for the probability distribution of positional errors incurred by geocoding residential addresses. In particular, we will formulate models that are sufficiently flexible to allow for the representation of features observed in empirical distributions of positional errors derived from a dataset of rural Iowa addresses, yet sufficiently simple that the aforementioned measurement-error and multiple imputation methodologies could be successfully implemented using these models. Positional errors corresponding to both automated geocoding and E911 geocoding will be considered. Upon formulating a suitable model or class of models for the errors, we will demonstrate how to fit those models to the data. Although the specific features seen in the distributions of positional errors from this predominantly rural Iowa county will not occur in all datasets, nor even in all error datasets derived from rural addresses, we believe that the methods we use to formulate and fit the models are generalizable to a great many datasets of positional errors incurred by geocoding.

## Methods

### Data

The address data upon which this investigation is based consist of all 2516 rural residential addresses in Carroll County, Iowa, USA, current as of 31 December 2005, which we obtained in conjunction with a comprehensive study of rural health in Iowa by the Iowa Department of Public Health and other researchers at the University of Iowa. A major objective of the study was to investigate the possible existence of associations between various health outcomes and exposure to environmental contaminants produced by concentrated animal feeding operations. Hence the focus on rural addresses, which were defined as all residential addresses that lie outside incorporated township boundaries.

### Geocodes and positional errors

An attempt was made to obtain a geocode of each rural address using an automated method, an E911 method, and an orthophoto method, as follows.

*Automated geocodes *were obtained by matching addresses to the U.S. Census Bureau's TIGER street centerline file for Carroll County using the GIS package ArcGIS 9.1 [23]. This process begins with automated parsing and standardization of the address list. Parsing is the process of breaking the address records up into distinct address component fields such as house number and street name, while standardization modifies these components, if necessary, so that they adhere to a common United States Postal Service standard [24]. Next, an address-ranged street segment in the TIGER file is probabilistically matched to each address on the basis of a "match score," which measures how closely each candidate address-ranged street segment in the TIGER file matches the address. Each field in the candidate segment is compared with the corresponding field of the address record being matched. The match score is a weighted composite score over all fields, scaled to lie between 0 to 100. For this analysis the minimum match score was set at either 100% (perfect matching) or 60%. Finally, the geocode is calculated by linearly interpolating the address number to a point on the matched street segment between the two points that define the limits of that segment's address range. No offset from the street centerline was used in this calculation so that the effect of not offsetting might show up in the positional error distribution.

As it happened, only 26 more addresses geocoded when a 60%-match criterion was used than when a 100%-match criterion was used, and of those additional geocodes, eight were extreme outliers occurring in three clusters located 12–16 km from their actual locations. A closer look at these outliers revealed that the extremely large positional errors were due to errors in the TIGER street centerline file such as an incorrect zip code, an address range for a street segment that fails to contain the house number, or a missing street segment. As a consequence of the automated geocoding software's matching algorithm, these errors tended to result in geocodes corresponding to an address with the same house number but lying on a street segment with a different but similar "name," e.g. "120th St" rather than "210th St," or "20th St" rather than "260th St." Rare, gregarious outliers such as these present a severe challenge to any modeling enterprise, including the mixture modeling approach to be featured here. Consequently, for our purposes we set these outliers aside and considered only the geocodes of 100%-matched addresses.

For emergency services dispatch purposes, *E911 geocodes *of all addresses in Carroll County are continually updated and maintained by the county government so that a 911 telephone caller within the county requesting assistance may be quickly and unambiguously located. The most suitable geocode for this purpose in rural areas was deemed by county officials to be the coordinates of the location where emergency service personnel would leave the public road and enter the private road leading to the property from which the call was made. We obtained these geocodes directly from the GIS coordinator of Carroll County, who was not able to say exactly how the contractor employed by Carroll County obtained them.

Using visual identification, the third author enhanced the E911 geocode for each address to a location centered on the residence related to the address. This task was accomplished with the aid of 24 inch/pixel grayscale orthophotos of the study area we obtained from the Carroll County GIS Administrator and color infrared orthophotos (with the same resolution) obtained from [25]. Hence we refer to this geocode as the *orthophoto-based geocode*. A GIS data layer indicating the parcel to which a particular property belonged (and which is used by the county assessor's office for tax assessment) was overlaid upon the orthophoto and E911 address layers to confirm that each geocode was assigned to the correct address.

Of the three geocoding methods, the orthophoto method is by far the most accurate, hence the geocodes produced by this method were taken as the "gold standard" or truth. For each of the other two methods, the positional error corresponding to a given address was determined as the vector difference of the address's geocode obtained by the method and that address's orthophoto-derived geocode. For various reasons – most frequently the inability to determine which of several buildings in the photograph was the residence – a completely reliable orthophoto-derived geocode could not be ascertained for 162 of the addresses, so our analysis of positional errors is based on the remaining 2354 addresses.

### Mixture models for the error distribution

In seeking useful models for a distribution of positional errors, one might first consider a bivariate normal distribution or a uniform distribution on a "standard" two-dimensional region (e.g. a circle or square). Indeed, normal and uniform distributions have been used previously to study the effects of location errors on spatial analyses in general, and on spatial prediction (kriging) and cluster detection in particular [26,16,19,20]. However, to the authors' knowledge no empirical evidence has ever been presented to demonstrate that these distributions adequately represent the probability distributions of positional errors corresponding to geocoded residential addresses. In fact, these relatively simple distributions will not be appropriate if, for instance, extremely large positional errors (outliers) occur more often than would be expected for a bivariate normal or uniform distribution, or if errors tend to cluster along more than one axial direction. It will be seen that outliers and "multi-axial clustering" both occur for the positional errors in our geocoded data, and thus simple normal or uniform distributions will not suffice. As alternatives, we propose the use of finite mixture distributions [27-29]. In a finite mixture distribution, each error can be regarded as having arisen from a population *G *which is a mixture of a finite number, say *g*, of subpopulations *G*_1_,..., *G*_*g *_in some proportions *p*_1_,..., *p*_*g*_, respectively, where ∑i=1gpi=1
 MathType@MTEF@5@5@+=feaafiart1ev1aaatCvAUfKttLearuWrP9MDH5MBPbIqV92AaeXatLxBI9gBaebbnrfifHhDYfgasaacH8akY=wiFfYdH8Gipec8Eeeu0xXdbba9frFj0=OqFfea0dXdd9vqai=hGuQ8kuc9pgc9s8qqaq=dirpe0xb9q8qiLsFr0=vr0=vr0dc8meaabaqaciaacaGaaeqabaqabeGadaaakeaadaaeWaqaaiabdchaWnaaBaaaleaacqWGPbqAaeqaaaqaaiabdMgaPjabg2da9iabigdaXaqaaiabdEgaNbqdcqGHris5aOGaeyypa0JaeGymaedaaa@383B@ and *p*_*i *_≥ 0 (*i *= 1,..., *g*). The probability density function (pdf) of an arbitrary positional error, **x**, can then be represented in the finite mixture form,

f(x;φ)=∑i=1gpifi(x;θ)     (1)
 MathType@MTEF@5@5@+=feaafiart1ev1aaatCvAUfKttLearuWrP9MDH5MBPbIqV92AaeXatLxBI9gBaebbnrfifHhDYfgasaacH8akY=wiFfYdH8Gipec8Eeeu0xXdbba9frFj0=OqFfea0dXdd9vqai=hGuQ8kuc9pgc9s8qqaq=dirpe0xb9q8qiLsFr0=vr0=vr0dc8meaabaqaciaacaGaaeqabaqabeGadaaakeaacqWGMbGzcqGGOaakieqacqWF4baEcqGG7aWoiiWacqGFgpGzcqGGPaqkcqGH9aqpdaaeWbqaaiabdchaWnaaBaaaleaacqWGPbqAaeqaaOGaemOzay2aaSbaaSqaaiabdMgaPbqabaGccqGGOaakcqWF4baEcqGG7aWocqGF4oqCcqGGPaqkaSqaaiabdMgaPjabg2da9iabigdaXaqaaiabdEgaNbqdcqGHris5aOGaaCzcaiaaxMaadaqadaqaaiabigdaXaGaayjkaiaawMcaaaaa@4B5C@

where *f*_*i*_(**x**; ***θ***) is the pdf corresponding to *G*_*i*_; ***θ ***denotes the vector of all unknown parameters associated with the parametric forms adopted for these *g *component pdfs; and ***φ ***= (**p'**, ***θ***')' where **p' **= (*p*_1_,..., *p*_*g*_). Furthermore, we focus on mixtures of bivariate normal and t distributions, which are the most commonly used mixture models for bivariate observations and are well-suited for observations contaminated by outliers and exhibiting multi-axial clustering. The t mixtures are more robust than normal mixtures to contamination by outliers, hence they generally yield more parsimonious models than normal mixtures for data with outliers.

### Estimation of parameters

For each of the two sets of positional errors – corresponding to automated and E911 geocodes – we obtained likelihood-based estimates of the parameters of normal mixtures and t mixtures for several values of *g*. For the normal mixtures, we estimated parameters using the method described by Basford and McLachlan [30], which is equivalent to applying the EM (expectation-maximization) algorithm [31] to this problem. A normal mixture has the form given by (1), with *i*th component pdf

fi(x;μi,Σi)=(2π)−1|Σi|−1/2exp⁡{−12(x−μi)′Σi−1(x−μi)}
 MathType@MTEF@5@5@+=feaafiart1ev1aaatCvAUfKttLearuWrP9MDH5MBPbIqV92AaeXatLxBI9gBaebbnrfifHhDYfgasaacH8akY=wiFfYdH8Gipec8Eeeu0xXdbba9frFj0=OqFfea0dXdd9vqai=hGuQ8kuc9pgc9s8qqaq=dirpe0xb9q8qiLsFr0=vr0=vr0dc8meaabaqaciaacaGaaeqabaqabeGadaaakeaacqWGMbGzdaWgaaWcbaGaemyAaKgabeaakiabcIcaOGqabiab=Hha4jabcUda7GGadiab+X7aTnaaBaaaleaacqWGPbqAaeqaaOGaeiilaWccceGae03Odm1aaSbaaSqaaiabdMgaPbqabaGccqGGPaqkcqGH9aqpcqGGOaakcqaIYaGmiiGacqaFapaCcqGGPaqkdaahaaWcbeqaaiabgkHiTiabigdaXaaakmaaemaabaGae03Odm1aaSbaaSqaaiabdMgaPbqabaaakiaawEa7caGLiWoadaahaaWcbeqaaiabgkHiTiabigdaXiabc+caViabikdaYaaakiGbcwgaLjabcIha4jabcchaWjabcUha7jabgkHiTmaalaaabaGaeGymaedabaGaeGOmaidaaiabcIcaOiab=Hha4jabgkHiTiab+X7aTnaaBaaaleaacqWGPbqAaeqaaOGafiykaKIbauaacqqFJoWudaqhaaWcbaGaemyAaKgabaGaeyOeI0IaeGymaedaaOGaeiikaGIae8hEaGNaeyOeI0Iae4hVd02aaSbaaSqaaiabdMgaPbqabaGccqGGPaqkcqGG9bqFaaa@6A55@

where ***μ***_*i *_and **Σ**_*i*_, are the mean vector and covariance matrix, respectively, of the *i*th component distribution. Thus, letting ***θ ***comprise **p**, ***μ***_1_,..., ***μ***_*g*_, and **Σ**_1_,..., **Σ**_*g*_, we find that the likelihood function corresponding to a random sample **x**_1_,..., **x**_*n *_from *G *is proportional to

L(φ)=∏j=1n∑i=1gpi|Σi|−1/2exp⁡{−12(xj−μi)′Σi−1(xj−μi)}.
 MathType@MTEF@5@5@+=feaafiart1ev1aaatCvAUfKttLearuWrP9MDH5MBPbIqV92AaeXatLxBI9gBaebbnrfifHhDYfgasaacH8akY=wiFfYdH8Gipec8Eeeu0xXdbba9frFj0=OqFfea0dXdd9vqai=hGuQ8kuc9pgc9s8qqaq=dirpe0xb9q8qiLsFr0=vr0=vr0dc8meaabaqaciaacaGaaeqabaqabeGadaaakeaacqWGmbatcqGGOaakiiWacqWFgpGzcqGGPaqkcqGH9aqpdaqeWbqaamaaqahabaGaemiCaa3aaSbaaSqaaiabdMgaPbqabaaabaGaemyAaKMaeyypa0JaeGymaedabaGaem4zaCganiabggHiLdaaleaacqWGQbGAcqGH9aqpcqaIXaqmaeaacqWGUbGBa0Gaey4dIunakmaaemaabaacceGae43Odm1aaSbaaSqaaiabdMgaPbqabaaakiaawEa7caGLiWoadaahaaWcbeqaaiabgkHiTiabigdaXiabc+caViabikdaYaaakiGbcwgaLjabcIha4jabcchaWjabcUha7jabgkHiTmaalaaabaGaeGymaedabaGaeGOmaidaaiabcIcaOGqabiab9Hha4naaBaaaleaacqWGQbGAaeqaaOGaeyOeI0Iae8hVd02aaSbaaSqaaiabdMgaPbqabaGccuGGPaqkgaqbaiab+n6atnaaDaaaleaacqWGPbqAaeaacqGHsislcqaIXaqmaaGccqGGOaakcqqF4baEdaWgaaWcbaGaemOAaOgabeaakiabgkHiTiab=X7aTnaaBaaaleaacqWGPbqAaeqaaOGaeiykaKIaeiyFa0NaeiOla4caaa@6EF6@

In this subsection the number of groups, *g*, is assumed to be known; methods for choosing *g *are deferred to the next subsection.

The likelihood equation,

*∂ *log *L *(*φ*)/*∂φ *= **0**,     (2)

is equivalent to the equations

p^i=∑j=1nw^ij/n,     (3)
 MathType@MTEF@5@5@+=feaafiart1ev1aaatCvAUfKttLearuWrP9MDH5MBPbIqV92AaeXatLxBI9gBaebbnrfifHhDYfgasaacH8akY=wiFfYdH8Gipec8Eeeu0xXdbba9frFj0=OqFfea0dXdd9vqai=hGuQ8kuc9pgc9s8qqaq=dirpe0xb9q8qiLsFr0=vr0=vr0dc8meaabaqaciaacaGaaeqabaqabeGadaaakeaacuWGWbaCgaqcamaaBaaaleaacqWGPbqAaeqaaOGaeyypa0ZaaabCaeaacuWG3bWDgaqcamaaBaaaleaacqWGPbqAcqWGQbGAaeqaaaqaaiabdQgaQjabg2da9iabigdaXaqaaiabd6gaUbqdcqGHris5aOGaei4la8IaemOBa4MaeiilaWIaaCzcaiaaxMaadaqadaqaaiabiodaZaGaayjkaiaawMcaaaaa@430C@

μ^i=∑j=1nw^ijxij/∑j=1nw^ij,     (4)
 MathType@MTEF@5@5@+=feaafiart1ev1aaatCvAUfKttLearuWrP9MDH5MBPbIqV92AaeXatLxBI9gBaebbnrfifHhDYfgasaacH8akY=wiFfYdH8Gipec8Eeeu0xXdbba9frFj0=OqFfea0dXdd9vqai=hGuQ8kuc9pgc9s8qqaq=dirpe0xb9q8qiLsFr0=vr0=vr0dc8meaabaqaciaacaGaaeqabaqabeGadaaakeaaiiWacuWF8oqBgaqcamaaBaaaleaacqWGPbqAaeqaaOGaeyypa0ZaaabCaeaacuWG3bWDgaqcamaaBaaaleaacqWGPbqAcqWGQbGAaeqaaGqabOGae4hEaG3aaSbaaSqaaiabdMgaPjabdQgaQbqabaaabaGaemOAaOMaeyypa0JaeGymaedabaGaemOBa4ganiabggHiLdGccqGGVaWldaaeWbqaaiqbdEha3zaajaWaaSbaaSqaaiabdMgaPjabdQgaQbqabaaabaGaemOAaOMaeyypa0JaeGymaedabaGaemOBa4ganiabggHiLdGccqGGSaalcaWLjaGaaCzcamaabmaabaGaeGinaqdacaGLOaGaayzkaaaaaa@51CE@

Σ^i=∑j=1nw^ij(xj−μ^i)(xj−μ^i)′/∑j=1nw^ij,     (5)
 MathType@MTEF@5@5@+=feaafiart1ev1aaatCvAUfKttLearuWrP9MDH5MBPbIqV92AaeXatLxBI9gBaebbnrfifHhDYfgasaacH8akY=wiFfYdH8Gipec8Eeeu0xXdbba9frFj0=OqFfea0dXdd9vqai=hGuQ8kuc9pgc9s8qqaq=dirpe0xb9q8qiLsFr0=vr0=vr0dc8meaabaqaciaacaGaaeqabaqabeGadaaakeaaiiqacuWFJoWugaqcamaaBaaaleaacqWGPbqAaeqaaOGaeyypa0ZaaabCaeaacuWG3bWDgaqcamaaBaaaleaacqWGPbqAcqWGQbGAaeqaaaqaaiabdQgaQjabg2da9iabigdaXaqaaiabd6gaUbqdcqGHris5aOGaeiikaGccbeGae4hEaG3aaSbaaSqaaiabdQgaQbqabaGccqGHsisliiWacuqF8oqBgaqcamaaBaaaleaacqWGPbqAaeqaaOGaeiykaKIaeiikaGIae4hEaG3aaSbaaSqaaiabdQgaQbqabaGccqGHsislcuqF8oqBgaqcamaaBaaaleaacqWGPbqAaeqaaOGafiykaKIbauaacqGGVaWldaaeWbqaaiqbdEha3zaajaWaaSbaaSqaaiabdMgaPjabdQgaQbqabaaabaGaemOAaOMaeyypa0JaeGymaedabaGaemOBa4ganiabggHiLdGccqGGSaalcaWLjaGaaCzcamaabmaabaGaeGynaudacaGLOaGaayzkaaaaaa@5F3E@

for *i *= 1,..., *g*, where

w^ij=p^i|Σ^i|−1/2exp⁡{−12(xj−μ^i)′Σ^i−1(xj−μ^i)}∑t=1gp^t|Σ^t|−1/2exp⁡{−12(xj−μ^t)′Σ^t−1(xj−μ^t)}.     (6)
 MathType@MTEF@5@5@+=feaafiart1ev1aaatCvAUfKttLearuWrP9MDH5MBPbIqV92AaeXatLxBI9gBaebbnrfifHhDYfgasaacH8akY=wiFfYdH8Gipec8Eeeu0xXdbba9frFj0=OqFfea0dXdd9vqai=hGuQ8kuc9pgc9s8qqaq=dirpe0xb9q8qiLsFr0=vr0=vr0dc8meaabaqaciaacaGaaeqabaqabeGadaaakeaacuWG3bWDgaqcamaaBaaaleaacqWGPbqAcqWGQbGAaeqaaOGaeyypa0ZaaSaaaeaacuWGWbaCgaqcamaaBaaaleaacqWGPbqAaeqaaOWaaqWaaeaaiiqacuWFJoWugaqcamaaBaaaleaacqWGPbqAaeqaaaGccaGLhWUaayjcSdWaaWbaaSqabeaacqGHsislcqaIXaqmcqGGVaWlcqaIYaGmaaGccyGGLbqzcqGG4baEcqGGWbaCcqGG7bWEcqGHsisldaWcaaqaaiabigdaXaqaaiabikdaYaaacqGGOaakieqacqGF4baEdaWgaaWcbaGaemOAaOgabeaakiabgkHiTGGadiqb9X7aTzaajaWaaSbaaSqaaiabdMgaPbqabaGccuGGPaqkgaqbaiqb=n6atzaajaWaa0baaSqaaiabdMgaPbqaaiabgkHiTiabigdaXaaakiabcIcaOiab+Hha4naaBaaaleaacqWGQbGAaeqaaOGaeyOeI0Iaf0hVd0MbaKaadaWgaaWcbaGaemyAaKgabeaakiabcMcaPiabc2ha9bqaamaaqadabaGafmiCaaNbaKaadaWgaaWcbaGaemiDaqhabeaaaeaacqWG0baDcqGH9aqpcqaIXaqmaeaacqWGNbWza0GaeyyeIuoakmaaemaabaGaf83OdmLbaKaadaWgaaWcbaGaemiDaqhabeaaaOGaay5bSlaawIa7amaaCaaaleqabaGaeyOeI0IaeGymaeJaei4la8IaeGOmaidaaOGagiyzauMaeiiEaGNaeiiCaaNaei4EaSNaeyOeI0YaaSaaaeaacqaIXaqmaeaacqaIYaGmaaGaeiikaGIae4hEaG3aaSbaaSqaaiabdQgaQbqabaGccqGHsislcuqF8oqBgaqcamaaBaaaleaacqWG0baDaeqaaOGafiykaKIbauaacuWFJoWugaqcamaaDaaaleaacqWG0baDaeaacqGHsislcqaIXaqmaaGccqGGOaakcqGF4baEdaWgaaWcbaGaemOAaOgabeaakiabgkHiTiqb9X7aTzaajaWaaSbaaSqaaiabdsha0bqabaGccqGGPaqkcqGG9bqFaaGaeiOla4IaaCzcaiaaxMaadaqadaqaaiabiAda2aGaayjkaiaawMcaaaaa@9AA5@

The w^ij
 MathType@MTEF@5@5@+=feaafiart1ev1aaatCvAUfKttLearuWrP9MDH5MBPbIqV92AaeXatLxBI9gBaebbnrfifHhDYfgasaacH8akY=wiFfYdH8Gipec8Eeeu0xXdbba9frFj0=OqFfea0dXdd9vqai=hGuQ8kuc9pgc9s8qqaq=dirpe0xb9q8qiLsFr0=vr0=vr0dc8meaabaqaciaacaGaaeqabaqabeGadaaakeaacuWG3bWDgaqcamaaBaaaleaacqWGPbqAcqWGQbGAaeqaaaaa@3117@ are weights such that w^ij
 MathType@MTEF@5@5@+=feaafiart1ev1aaatCvAUfKttLearuWrP9MDH5MBPbIqV92AaeXatLxBI9gBaebbnrfifHhDYfgasaacH8akY=wiFfYdH8Gipec8Eeeu0xXdbba9frFj0=OqFfea0dXdd9vqai=hGuQ8kuc9pgc9s8qqaq=dirpe0xb9q8qiLsFr0=vr0=vr0dc8meaabaqaciaacaGaaeqabaqabeGadaaakeaacuWG3bWDgaqcamaaBaaaleaacqWGPbqAcqWGQbGAaeqaaaaa@3117@ is an estimate of the probability that observation *j *belongs to component group *i*. Equations (3)-(6) can be solved iteratively upon first making an initial assignment of observations to groups and supplying an initial estimate of ***φ ***to (6), and then iterating until convergence. The resulting estimate of ***φ ***is a solution to (2) and is thus a local maximum of *L*(***φ***). However, it is generally not a global maximum; in fact, (2) has multiple roots, and *L*(***φ***) is unbounded so the maximum likelihood estimator of ***φ ***does not exist [32]. Nevertheless, for mixtures of univariate normals it is known that the sequence of roots of (2) corresponding to the largest of the local maxima is consistent, asymptotically normal, and efficient [33], and the same result is widely believed to hold for mixtures of bivariate normals as well. We refer to the root corresponding to the largest of the local maxima as the likelihood-based estimate. To increase the prospects of finding the largest of the local maxima, it is recommended that the iterative solution process begin from several different initial values. The *j*th observation may be given a final assignment to a group on the basis of the maximum of the converged w^ij
 MathType@MTEF@5@5@+=feaafiart1ev1aaatCvAUfKttLearuWrP9MDH5MBPbIqV92AaeXatLxBI9gBaebbnrfifHhDYfgasaacH8akY=wiFfYdH8Gipec8Eeeu0xXdbba9frFj0=OqFfea0dXdd9vqai=hGuQ8kuc9pgc9s8qqaq=dirpe0xb9q8qiLsFr0=vr0=vr0dc8meaabaqaciaacaGaaeqabaqabeGadaaakeaacuWG3bWDgaqcamaaBaaaleaacqWGPbqAcqWGQbGAaeqaaaaa@3117@ across *i*.

The normal mixture likelihood-based estimation method just described was carried out for the Carroll County positional error data using the FORTRAN program EMMIX written by D. Peel and G.J. McLachlan, which can be downloaded freely from [34]. To obtain the initial classification of the data needed for starting the estimation algorithm, the data were partitioned randomly into *g *groups 50 times, and the partition that produced the highest likelihood was adopted as the initial classification. The proportion of observations belonging to the *i*th group in this initial classification was taken as the initial estimate of *p*_*i*_, and the sample mean vector and sample covariance matrix of the observations belonging to the *i*th group were taken an initial estimates of ***μ***_*i *_and **Σ**_*i*_, respectively.

For the t mixture models, we obtained likelihood-based estimates of parameters using the ECM (expectation-conditional maximization) method described by McLachlan and Krishnan [35]. The *i*th component pdf of a t mixture is of the form

fi(x;μi,Σi,νi)=Γ(1+νi2)|Σi|−1/2πνiΓ(νi/2){1+(x−μi)′Σi−1(x−μi)/νi}1+νi/2     (7)
 MathType@MTEF@5@5@+=feaafiart1ev1aaatCvAUfKttLearuWrP9MDH5MBPbIqV92AaeXatLxBI9gBaebbnrfifHhDYfgasaacH8akY=wiFfYdH8Gipec8Eeeu0xXdbba9frFj0=OqFfea0dXdd9vqai=hGuQ8kuc9pgc9s8qqaq=dirpe0xb9q8qiLsFr0=vr0=vr0dc8meaabaqaciaacaGaaeqabaqabeGadaaakeaacqWGMbGzdaWgaaWcbaGaemyAaKgabeaakiabcIcaOGqabiab=Hha4jabcUda7GGadiab+X7aTnaaBaaaleaacqWGPbqAaeqaaOGaeiilaWccceGae03Odm1aaSbaaSqaaiabdMgaPbqabaGccqGGSaaliiGacqaF9oGBdaWgaaWcbaGaemyAaKgabeaakiabcMcaPiabg2da9maalaaabaGaeu4KdCKaeiikaGIaeGymaeJaey4kaSYaaSaaaeaacqaF9oGBdaWgaaWcbaGaemyAaKgabeaaaOqaaiabikdaYaaacqGGPaqkdaabdaqaaiab9n6atnaaBaaaleaacqWGPbqAaeqaaaGccaGLhWUaayjcSdWaaWbaaSqabeaacqGHsislcqaIXaqmcqGGVaWlcqaIYaGmaaaakeaacqaFapaCcqaF9oGBdaWgaaWcbaGaemyAaKgabeaakiabfo5ahjabcIcaOiab817aUnaaBaaaleaacqWGPbqAaeqaaOGaei4la8IaeGOmaiJaeiykaKIaei4EaSNaeGymaeJaey4kaSIaeiikaGIae8hEaGNaeyOeI0Iae4hVd02aaSbaaSqaaiabdMgaPbqabaGccuGGPaqkgaqbaiab9n6atnaaDaaaleaacqWGPbqAaeaacqGHsislcqaIXaqmaaGccqGGOaakcqWF4baEcqGHsislcqGF8oqBdaWgaaWcbaGaemyAaKgabeaakiabcMcaPiabc+caViab817aUnaaBaaaleaacqWGPbqAaeqaaOGaeiyFa03aaWbaaSqabeaacqaIXaqmcqGHRaWkcqaF9oGBdaWgaaadbaGaemyAaKgabeaaliabc+caViabikdaYaaaaaGccaWLjaGaaCzcamaabmaabaGaeG4naCdacaGLOaGaayzkaaaaaa@8844@

where Γ(·) is the gamma function, and ***μ***_*i *_and **Σ**_*i*_ are the mean vector and covariance matrix, respectively, and *v*_*i *_is the degrees of freedom parameter, of the *i*th component distribution. The degrees of freedom may be viewed as a robustness (to outliers) tuning parameter: a component t pdf with small *v *has heavy tails, but as *v *tends to infinity the tails become lighter and the corresponding t component pdf tends to a normal pdf. The likelihood function corresponding to a random sample **x**_1_,..., **x**_*n *_from a *g*-component t mixture *G *is then given by

L(φ)=∏j=1n∑i=1gpifi(xj:μi,Σi,νi),
 MathType@MTEF@5@5@+=feaafiart1ev1aaatCvAUfKttLearuWrP9MDH5MBPbIqV92AaeXatLxBI9gBaebbnrfifHhDYfgasaacH8akY=wiFfYdH8Gipec8Eeeu0xXdbba9frFj0=OqFfea0dXdd9vqai=hGuQ8kuc9pgc9s8qqaq=dirpe0xb9q8qiLsFr0=vr0=vr0dc8meaabaqaciaacaGaaeqabaqabeGadaaakeaacqWGmbatcqGGOaakiiWacqWFgpGzcqGGPaqkcqGH9aqpdaqeWbqaamaaqahabaGaemiCaa3aaSbaaSqaaiabdMgaPbqabaGccqWGMbGzdaWgaaWcbaGaemyAaKgabeaaaeaacqWGPbqAcqGH9aqpcqaIXaqmaeaacqWGNbWza0GaeyyeIuoaaSqaaiabdQgaQjabg2da9iabigdaXaqaaiabd6gaUbqdcqGHpis1aOGaeiikaGccbeGae4hEaG3aaSbaaSqaaiabdQgaQbqabaGccqGG6aGocqWF8oqBdaWgaaWcbaGaemyAaKgabeaakiabcYcaSGGabiab9n6atnaaBaaaleaacqWGPbqAaeqaaOGaeiilaWccciGaeWxVd42aaSbaaSqaaiabdMgaPbqabaGccqGGPaqkcqGGSaalaaa@57F4@

with *f*_*i*_(·) defined in (7) and with ***φ ***comprising *p*_1_,..., *p*_*g*_, ***μ***_1_,..., ***μ***_*g*_, **Σ**_1_,..., **Σ**_*g*_, and *v*_1_,..., *v*_*g*_. Details of the implementation of the ECM estimation algorithm to t mixture models are too lengthy to report here; however, they can be found in [36]. The algorithm was implemented for the Carroll County positional error data using the same program that was used to fit normal mixtures, viz. EMMIX, and the same random grouping scheme used for normal mixtures was used to initially classify the data and obtain initial parameter estimates.

### Choosing the number of components

In the previous subsection it was assumed that the number of components in the mixture distribution was known. While this assumption is appropriate for some applications of mixture models, for example when the subpopulations are males and females or a known number of age classes, it is generally not appropriate for modeling positional errors incurred by geocoding. Thus, the number of components in a mixture distribution for positional errors must be determined using the data at hand. Several methods for accomplishing this have been proposed, ranging from informal graphical techniques to more formal hypothesis testing procedures. Here, we choose the number of components using the *BIC *(Bayesian Information Criterion), a commonly-used model selection method less formal than hypothesis testing but more formal than mere graphical analysis [37]. For a model with *k *parameters to be estimated, *BIC *is given by

*BIC *= -2 log *L *(φ^
 MathType@MTEF@5@5@+=feaafiart1ev1aaatCvAUfKttLearuWrP9MDH5MBPbIqV92AaeXatLxBI9gBaebbnrfifHhDYfgasaacH8akY=wiFfYdH8Gipec8Eeeu0xXdbba9frFj0=OqFfea0dXdd9vqai=hGuQ8kuc9pgc9s8qqaq=dirpe0xb9q8qiLsFr0=vr0=vr0dc8meaabaqaciaacaGaaeqabaqabeGadaaakeaaiiGacuWFgpGzgaqcaaaa@2E7C@) + *k *log *n*

where L(φ^
 MathType@MTEF@5@5@+=feaafiart1ev1aaatCvAUfKttLearuWrP9MDH5MBPbIqV92AaeXatLxBI9gBaebbnrfifHhDYfgasaacH8akY=wiFfYdH8Gipec8Eeeu0xXdbba9frFj0=OqFfea0dXdd9vqai=hGuQ8kuc9pgc9s8qqaq=dirpe0xb9q8qiLsFr0=vr0=vr0dc8meaabaqaciaacaGaaeqabaqabeGadaaakeaaiiGacuWFgpGzgaqcaaaa@2E7C@) is the likelihood function for the *n *observations, evaluated at the likelihood-based estimator φ^
 MathType@MTEF@5@5@+=feaafiart1ev1aaatCvAUfKttLearuWrP9MDH5MBPbIqV92AaeXatLxBI9gBaebbnrfifHhDYfgasaacH8akY=wiFfYdH8Gipec8Eeeu0xXdbba9frFj0=OqFfea0dXdd9vqai=hGuQ8kuc9pgc9s8qqaq=dirpe0xb9q8qiLsFr0=vr0=vr0dc8meaabaqaciaacaGaaeqabaqabeGadaaakeaaiiGacuWFgpGzgaqcaaaa@2E7C@. *BIC *combines a measure of badness-of-fit, -2 log *L*(φ^
 MathType@MTEF@5@5@+=feaafiart1ev1aaatCvAUfKttLearuWrP9MDH5MBPbIqV92AaeXatLxBI9gBaebbnrfifHhDYfgasaacH8akY=wiFfYdH8Gipec8Eeeu0xXdbba9frFj0=OqFfea0dXdd9vqai=hGuQ8kuc9pgc9s8qqaq=dirpe0xb9q8qiLsFr0=vr0=vr0dc8meaabaqaciaacaGaaeqabaqabeGadaaakeaaiiGacuWFgpGzgaqcaaaa@2E7C@), with a measure of model complexity, *k *log *n*. When comparing two models, the model with the smaller *BIC *is to be preferred, apart from any other considerations. In the present context, however, we value model parsimony even more highly than usual because of the compelling need for simplicity in measurement-error modeling approaches for handling location uncertainty in spatial analyses. Therefore, although we will use *BIC *as a guide for model selection, we may prefer a model with a slightly larger *BIC *than another if it is considerably more parsimonious.

### Mixture modeling example

**Figure 1:**
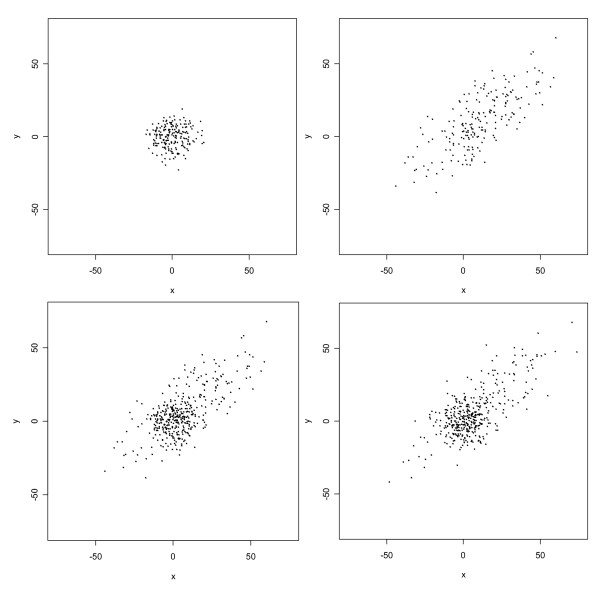
Scatterplot of simulated data from two-component bivariate normal mixture model. The upper left panel displays 200 observations from the first component; the upper right panel displays 200 observations from the second component; the lower left panel is a superposition of the two upper panels; and the lower right panel displays a new simulation of 400 observations from the two-component normal mixture model fitted to the data from the original simulation.

We provide the following example to illustrate the effectiveness of the mixture model estimation and model selection methodology. Two hundred observations were simulated from a bivariate normal distribution with means *μ*_*X *_= *μ*_*Y *_= 0 (for both variables), variances σX2
 MathType@MTEF@5@5@+=feaafiart1ev1aaatCvAUfKttLearuWrP9MDH5MBPbIqV92AaeXatLxBI9gBaebbnrfifHhDYfgasaacH8akY=wiFfYdH8Gipec8Eeeu0xXdbba9frFj0=OqFfea0dXdd9vqai=hGuQ8kuc9pgc9s8qqaq=dirpe0xb9q8qiLsFr0=vr0=vr0dc8meaabaqaciaacaGaaeqabaqabeGadaaakeaaiiGacqWFdpWCdaqhaaWcbaGaemiwaGfabaGaeGOmaidaaaaa@30CE@ = σY2
 MathType@MTEF@5@5@+=feaafiart1ev1aaatCvAUfKttLearuWrP9MDH5MBPbIqV92AaeXatLxBI9gBaebbnrfifHhDYfgasaacH8akY=wiFfYdH8Gipec8Eeeu0xXdbba9frFj0=OqFfea0dXdd9vqai=hGuQ8kuc9pgc9s8qqaq=dirpe0xb9q8qiLsFr0=vr0=vr0dc8meaabaqaciaacaGaaeqabaqabeGadaaakeaaiiGacqWFdpWCdaqhaaWcbaGaemywaKfabaGaeGOmaidaaaaa@30D0@ = 64, and correlation coefficient *ρ *= 0; and another 200 observations were simulated from a bivariate normal distribution with means *μ*_*X *_= *μ*_*Y *_= 10, variances σX2
 MathType@MTEF@5@5@+=feaafiart1ev1aaatCvAUfKttLearuWrP9MDH5MBPbIqV92AaeXatLxBI9gBaebbnrfifHhDYfgasaacH8akY=wiFfYdH8Gipec8Eeeu0xXdbba9frFj0=OqFfea0dXdd9vqai=hGuQ8kuc9pgc9s8qqaq=dirpe0xb9q8qiLsFr0=vr0=vr0dc8meaabaqaciaacaGaaeqabaqabeGadaaakeaaiiGacqWFdpWCdaqhaaWcbaGaemiwaGfabaGaeGOmaidaaaaa@30CE@ = σY2
 MathType@MTEF@5@5@+=feaafiart1ev1aaatCvAUfKttLearuWrP9MDH5MBPbIqV92AaeXatLxBI9gBaebbnrfifHhDYfgasaacH8akY=wiFfYdH8Gipec8Eeeu0xXdbba9frFj0=OqFfea0dXdd9vqai=hGuQ8kuc9pgc9s8qqaq=dirpe0xb9q8qiLsFr0=vr0=vr0dc8meaabaqaciaacaGaaeqabaqabeGadaaakeaaiiGacqWFdpWCdaqhaaWcbaGaemywaKfabaGaeGOmaidaaaaa@30D0@ = 400, and correlation coefficient *ρ *= 0.75. Each group of observations and their superposition is displayed in Figure 1 (upper panels and lower left panel). Normal mixture models with *g *= 1, 2, 3,4, or 5 components were fit to these data using EMMIX. Values of *BIC *for these fitted models were 6469, 6387, 6408, 6420, and 6442, respectively. Thus, the two-component model fits best, as it should. For the two-component model, likelihood-based parameter estimates were as follows:

First component: p^
 MathType@MTEF@5@5@+=feaafiart1ev1aaatCvAUfKttLearuWrP9MDH5MBPbIqV92AaeXatLxBI9gBaebbnrfifHhDYfgasaacH8akY=wiFfYdH8Gipec8Eeeu0xXdbba9frFj0=OqFfea0dXdd9vqai=hGuQ8kuc9pgc9s8qqaq=dirpe0xb9q8qiLsFr0=vr0=vr0dc8meaabaqaciaacaGaaeqabaqabeGadaaakeaacuWGWbaCgaqcaaaa@2E25@ = 0.53, μ^
 MathType@MTEF@5@5@+=feaafiart1ev1aaatCvAUfKttLearuWrP9MDH5MBPbIqV92AaeXatLxBI9gBaebbnrfifHhDYfgasaacH8akY=wiFfYdH8Gipec8Eeeu0xXdbba9frFj0=OqFfea0dXdd9vqai=hGuQ8kuc9pgc9s8qqaq=dirpe0xb9q8qiLsFr0=vr0=vr0dc8meaabaqaciaacaGaaeqabaqabeGadaaakeaaiiGacuWF8oqBgaqcaaaa@2E79@ = 0.3, μ^
 MathType@MTEF@5@5@+=feaafiart1ev1aaatCvAUfKttLearuWrP9MDH5MBPbIqV92AaeXatLxBI9gBaebbnrfifHhDYfgasaacH8akY=wiFfYdH8Gipec8Eeeu0xXdbba9frFj0=OqFfea0dXdd9vqai=hGuQ8kuc9pgc9s8qqaq=dirpe0xb9q8qiLsFr0=vr0=vr0dc8meaabaqaciaacaGaaeqabaqabeGadaaakeaaiiGacuWF8oqBgaqcaaaa@2E79@ = -0.5, σ^X2
 MathType@MTEF@5@5@+=feaafiart1ev1aaatCvAUfKttLearuWrP9MDH5MBPbIqV92AaeXatLxBI9gBaebbnrfifHhDYfgasaacH8akY=wiFfYdH8Gipec8Eeeu0xXdbba9frFj0=OqFfea0dXdd9vqai=hGuQ8kuc9pgc9s8qqaq=dirpe0xb9q8qiLsFr0=vr0=vr0dc8meaabaqaciaacaGaaeqabaqabeGadaaakeaaiiGacuWFdpWCgaqcamaaDaaaleaacqWGybawaeaacqaIYaGmaaaaaa@30DE@ = 55.7, σ^Y2
 MathType@MTEF@5@5@+=feaafiart1ev1aaatCvAUfKttLearuWrP9MDH5MBPbIqV92AaeXatLxBI9gBaebbnrfifHhDYfgasaacH8akY=wiFfYdH8Gipec8Eeeu0xXdbba9frFj0=OqFfea0dXdd9vqai=hGuQ8kuc9pgc9s8qqaq=dirpe0xb9q8qiLsFr0=vr0=vr0dc8meaabaqaciaacaGaaeqabaqabeGadaaakeaaiiGacuWFdpWCgaqcamaaDaaaleaacqWGzbqwaeaacqaIYaGmaaaaaa@30E0@ = 60.3, ρ^
 MathType@MTEF@5@5@+=feaafiart1ev1aaatCvAUfKttLearuWrP9MDH5MBPbIqV92AaeXatLxBI9gBaebbnrfifHhDYfgasaacH8akY=wiFfYdH8Gipec8Eeeu0xXdbba9frFj0=OqFfea0dXdd9vqai=hGuQ8kuc9pgc9s8qqaq=dirpe0xb9q8qiLsFr0=vr0=vr0dc8meaabaqaciaacaGaaeqabaqabeGadaaakeaaiiGacuWFbpGCgaqcaaaa@2E83@ = 0.01

Second component: p^
 MathType@MTEF@5@5@+=feaafiart1ev1aaatCvAUfKttLearuWrP9MDH5MBPbIqV92AaeXatLxBI9gBaebbnrfifHhDYfgasaacH8akY=wiFfYdH8Gipec8Eeeu0xXdbba9frFj0=OqFfea0dXdd9vqai=hGuQ8kuc9pgc9s8qqaq=dirpe0xb9q8qiLsFr0=vr0=vr0dc8meaabaqaciaacaGaaeqabaqabeGadaaakeaacuWGWbaCgaqcaaaa@2E25@ = 0.47, μ^
 MathType@MTEF@5@5@+=feaafiart1ev1aaatCvAUfKttLearuWrP9MDH5MBPbIqV92AaeXatLxBI9gBaebbnrfifHhDYfgasaacH8akY=wiFfYdH8Gipec8Eeeu0xXdbba9frFj0=OqFfea0dXdd9vqai=hGuQ8kuc9pgc9s8qqaq=dirpe0xb9q8qiLsFr0=vr0=vr0dc8meaabaqaciaacaGaaeqabaqabeGadaaakeaaiiGacuWF8oqBgaqcaaaa@2E79@ = 10.9, μ^
 MathType@MTEF@5@5@+=feaafiart1ev1aaatCvAUfKttLearuWrP9MDH5MBPbIqV92AaeXatLxBI9gBaebbnrfifHhDYfgasaacH8akY=wiFfYdH8Gipec8Eeeu0xXdbba9frFj0=OqFfea0dXdd9vqai=hGuQ8kuc9pgc9s8qqaq=dirpe0xb9q8qiLsFr0=vr0=vr0dc8meaabaqaciaacaGaaeqabaqabeGadaaakeaaiiGacuWF8oqBgaqcaaaa@2E79@ = 11.5, σ^X2
 MathType@MTEF@5@5@+=feaafiart1ev1aaatCvAUfKttLearuWrP9MDH5MBPbIqV92AaeXatLxBI9gBaebbnrfifHhDYfgasaacH8akY=wiFfYdH8Gipec8Eeeu0xXdbba9frFj0=OqFfea0dXdd9vqai=hGuQ8kuc9pgc9s8qqaq=dirpe0xb9q8qiLsFr0=vr0=vr0dc8meaabaqaciaacaGaaeqabaqabeGadaaakeaaiiGacuWFdpWCgaqcamaaDaaaleaacqWGybawaeaacqaIYaGmaaaaaa@30DE@ = 446.8, σ^Y2
 MathType@MTEF@5@5@+=feaafiart1ev1aaatCvAUfKttLearuWrP9MDH5MBPbIqV92AaeXatLxBI9gBaebbnrfifHhDYfgasaacH8akY=wiFfYdH8Gipec8Eeeu0xXdbba9frFj0=OqFfea0dXdd9vqai=hGuQ8kuc9pgc9s8qqaq=dirpe0xb9q8qiLsFr0=vr0=vr0dc8meaabaqaciaacaGaaeqabaqabeGadaaakeaaiiGacuWFdpWCgaqcamaaDaaaleaacqWGzbqwaeaacqaIYaGmaaaaaa@30E0@ = 367.9, ρ^
 MathType@MTEF@5@5@+=feaafiart1ev1aaatCvAUfKttLearuWrP9MDH5MBPbIqV92AaeXatLxBI9gBaebbnrfifHhDYfgasaacH8akY=wiFfYdH8Gipec8Eeeu0xXdbba9frFj0=OqFfea0dXdd9vqai=hGuQ8kuc9pgc9s8qqaq=dirpe0xb9q8qiLsFr0=vr0=vr0dc8meaabaqaciaacaGaaeqabaqabeGadaaakeaaiiGacuWFbpGCgaqcaaaa@2E83@ = 0.75.

These estimates match the true parameter values very well. Finally, the fitted mixture model was used to generate a new set of 400 observations, which are also displayed in Figure 1 (lower right panel). Upon comparing this display with that for the original set of observations, we see that the fitted model generates data that closely resemble the original simulated data. In this sense, then, the fitted model has excellent predictive power.

## Results and Discussion

### Automated geocoding errors

**Figure 2:**
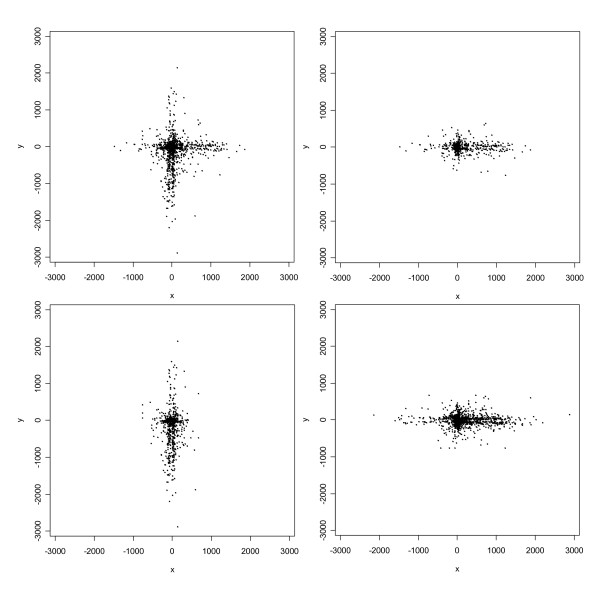
Scatterplot of positional errors (in meters) for the automated geocodes. The upper left panel displays the complete data; the upper right panel displays errors for addresses on streets aligned E-W; the lower left panel displays errors for addresses on streets aligned N-S; and the lower right panel is a superposition of the upper right panel and a 90-degree counterclockwise rotation of the lower left panel.

Of the 2354 rural addresses in Carroll County with orthophoto-derived geocodes, 1423 (60.5%) geocoded using the automated method with a 100%-match criterion. The positional errors (which are two-dimensional vectors) associated with these geocodes ranged in length from a minimum of 3 m to a maximum of 2896 m, with a median of 168 m, and are displayed as points in Figure 2. Interestingly, the errors tend to cluster along the N-S and E-W axial directions in such a way that the overall shape of their distribution, apart from a few outliers, resembles a Greek cross (Figure 2, upper left panel). More errors lie near the center of the cross than near its extremities. Moreover, there is a distinct shift in the mean with respect to the origin along each axial direction: along the E-W axis many more errors occur to the east of zero, while along the N-S axis many more errors occur to the south of zero. Close scrutiny also indicates the existence of two parallel "strands" of errors along each axial direction, which straddle the axes and are likely due to relatively small offsets of residences from street centerlines. Still more interesting features become apparent upon plotting the errors for the 662 addresses on streets running mainly E-W separately from the errors for the 761 addresses on streets running mainly N-S (Figure 2, upper right and lower left panels). This decomposition shows that while the errors near the cross's center appear to be relatively isotropic, i.e. occurring more or less equally often in all directions, those errors away from the center tend to be aligned with the axial orientation of the street on which the corresponding address lies.

Manual checking of the fifty largest errors revealed that many were attributable to street segments in the TIGER/Line file that had correct street names but incorrect address ranges. Others appeared to be attributable to interpolation errors or possibly house address numbering "errors" (i.e. deviations from the distance-from-intersection rule or some other rule that was used when the houses were originally numbered). These database and procedural errors, in combination with the high degree of rectilinearity of the rural road network in Carroll County, produce the distinctive Greek-cross shape of the empirical distribution of positional errors. Outliers from this overall shape appear to be due to either very large offsets (e.g., one house was nearly 800 m from its corresponding street centerline), incorrect TIGER/Line file geometry, or both.

We do not have a ready explanation for the bias with respect to the origin exhibited by the errors. However, the fact that the mean errors are shifted to the east along E-W streets and south along N-S streets, in tandem with the fact that these directions of shift coincide with the directions in which rural house numbers are ascending, suggest that the explanation has something to do with a systematic interpolation or house numbering procedural error. As a follow-up, we computed the mean error for each individual street and found that these means were consistently, in fact invariably, to the east and south. Thus the bias is pervasive, not merely limited to a few streets.

Owing to the Greek-cross shape of the empirical distribution of the entire set of positional errors, no single bivariate normal or t distribution will fit them well, nor for that matter will *any *elliptical distribution (i.e. a distribution whose contours of equal probability are ellipses). However, the decay in frequency of points with increasing distance from a central location along each axis suggests that a mixture of two or more normal or t distributions, of which at least one is aligned in approximately a N-S direction and at least one other is aligned in approximately an E-W direction, might provide an adequate fit. Consequently, normal and t mixtures with various numbers of components were fit to the errors. Values of *BIC *for each mixture model are given in Table 1a. The results indicate that a three-component mixture fits much better than a two-component mixture, but increasing the number of components beyond three results in marginal improvement in fit. The results also show the t mixture model to be superior to the normal mixture model. In light of these results and taking into account the premium on simplicity in measurement-error models, we would select the three-component t model for these errors.

**Table 1:** Bayesian Information Criteria (*BIC*) for normal and t mixture models.

Error dataset	Distribution	Number of Components	*BIC*
(a)	Normal	1	48103
	Normal	2	45851
	Normal	3	45236
	Normal	4	45124
	t	1	46083
	t	2	45358
	t	3	45056
	t	4	45042

(b)	Normal	1	46422
	Normal	2	44809
	Normal	3	44597
	Normal	4	44557
	t	1	45659
	t	2	44538
	t	3	44516
	t	4	44459

(c)	Normal	1	67174
	Normal	2	63174
	Normal	3	62710
	Normal	4	62446
	t	1	62841
	t	2	62345
	t	3	62219
	t	4	62230

(d)	Normal	1	64227
	Normal	2	61360
	Normal	3	61101
	Normal	4	61059
	t	1	61092
	t	2	60980
	t	3	60982
	t	4	60994

Models with several different numbers of components, were fitted to the following four error datasets: (a) automated geocoding positional errors; (b) automated geocoding positional errors aligned with axial direction of corresponding street segment; (c) E911 positional errors; (d) E911 positional errors aligned with axial direction of corresponding street segment.

**Figure 3:**
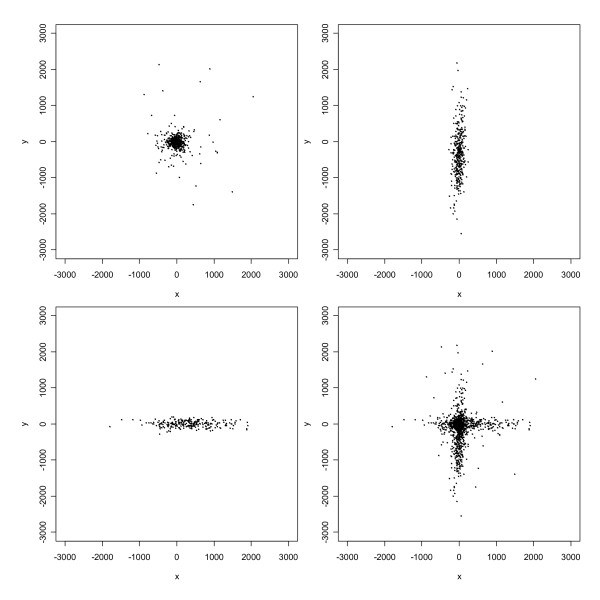
Simulated data from the fitted three-component t mixture distribution for the automated geocoding errors. The upper left panel, upper right panel, and lower left panel correspond to components in order of decreasing *p*_*i*_; and the lower right panel is their superposition.

Likelihood-based estimates of the mean vector and covariance matrix for the three-component t model are given in Table 2a, and Figure 3 depicts 1423 simulated observations from the fitted model. (The number of simulated observations was chosen to match the number of real observations so that plots would be directly comparable.) Upon comparing the lower right panel of Figure 3 with the upper left panel of Figure 2, we see that the fitted model reproduces the large-scale features of the positional errors quite well. Furthermore, the parameter estimates and component plots indicate that: (1) the largest component group consists of errors which are mostly "small" (less than 100 m), relatively isotropic, and centered at the origin, but heavy-tailed (*v *= 1.6) and thus including some outliers; (2) the other two component groups, comprising relative proportions roughly equivalent to the relative numbers of addresses on N-S and E-W streets, respectively, include many errors of intermediate to relatively large size (> 500 m), which are aligned in the N-S and E-W axial directions, respectively, but are lighter-tailed (*v *= 6.5 and *v *= 19.6) than the first component and hence relatively devoid of outliers; and (3) the means of the second and third components are several hundred meters to the east and south, respectively, of the origin, which is consistent with the systematic bias in these directions noted previously.

**Table 2:** Likelihood-based parameter estimates for the best-fitting models.

Error dataset	Component	Proportion	*μ*_*X*_	*μ*_*Y*_	*σ*_*X*_	*σ*_*Y*_	*ρ*	*v*
(a)	1	0.571	-12.1	-10.7	61.6	54.1	-0.05	1.6
	2	0.253	-4.7	-350.0	75.9	550.0	0.18	6.5
	3	0.176	352.8	-12.6	540.3	84.9	-0.03	16.7
(b)	1	0.560	-0.8	-14.2	39.4	75.9	0.06	1.8
	2	0.440	372.1	-6.7	523.6	90.3	-0.10	5.9
(c)	1	0.519	4.9	-5.4	62.3	60.8	-0.10	1.8
	2	0.292	13.6	-35.0	289.1	54.9	-0.14	2.4
	3	0.189	14.9	-10.2	62.1	354.4	0.14	2.4
(d)	1	0.700	5.9	-4.3	47.0	100.7	0.06	1.8
	2	0.300	29.3	-6.2	62.1	419.5	0.16	3.0

Models and the datasets to which they were fitted are: (a) the three-component t mixture model for the automated geocoding positional errors; (b) the two-component t mixture model for the automated geocoding positional errors aligned with axial direction of corresponding street segment; (c) the three-component t mixture model for the E911 positional errors; (d) the two-component t mixture model for the E911 positional errors aligned with axial direction of corresponding street segment. Means are denoted by *μ*_*X *_and *μ*_*Y*_, standard deviations by *σ*_*X *_and *σ*_*Y*_, correlation coefficient by *ρ*, and degrees of freedom by *v*. Units of measurement for means and standard deviations are meters.

The lower right panel of Figure 2 displays the "aligned errors," i.e. the errors relative to the axial orientation of the street segment on which the corresponding address lies. Equivalently, the aligned errors are a superposition of the points in the upper right panel and those resulting from a 90-degree counterclockwise rotation of the lower left panel of the same figure. Normal and t mixtures were also fitted to the aligned errors. Values of *BIC *and likelihood-based parameter estimates are given in Tables 1b and 2b, respectively. The results suggest that a two-component t mixture fits adequately well; that the first component of this mixture is essentially the same as the first component of the three-component t mixture for the original errors; and that the second component is essentially the combination of the third component and rotated second component of the three-component t mixture for the original errors. In fact, *BIC *for the two-component t mixture for the aligned errors is substantially smaller than *BIC *for the three-component t mixture for the original errors (Table 1), which indicates that accounting for the orientation of the street on which an address lies results in a more parsimonious model with no reduction in model adequacy.

### E911 geocoding errors

**Figure 4:**
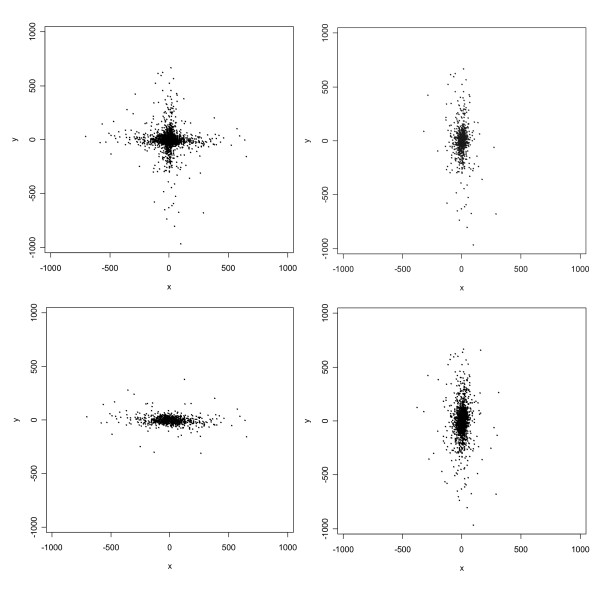
Scatterplot of the positional errors (in meters) for the E911 geocodes. The upper left panel displays the complete data; The upper right panel displays errors for addresses on streets aligned E-W; The lower left panel displays errors for addresses on streets aligned N-S; and the lower right panel is a superposition of the upper right panel and a 90-degree counterclockwise rotation of the lower left panel.

The positional errors corresponding to the 2354 E911 geocodes (Figure 4) ranged in length from 2 m to 974 m, with a median of 44 m. Thus, these errors tend to be considerably smaller than their automated geocoding counterparts. The upper left panel of Figure 4 shows the errors to be arrayed in a Greek cross-like configuration that appears even more pronounced than was the case for the automated geocoding errors, so likewise a single normal or t distribution will not fit well. But once again there is an attenuation in the frequency of points with increasing distance from a central point along each axis, suggesting that a mixture of two or more normal or t distributions might fit the data well. Moreover, the aforementioned central point of the distribution appears to be at or very close to the origin; there is not a mean shift with respect to the origin along each axis as there was for the automated geocoding errors. Nor do there appear to be "strands" of points straddling, and running parallel to, each coordinate axis, as there were for the automated geocoding errors. However, there are outliers, and there is an interesting effect of orientational alignment: upon plotting the 1116 addresses on streets aligned mainly E-W separately from the 1238 addresses on streets aligned mainly N-S (Figure 4, upper right and lower left panels), we observe that the errors tend to be aligned *orthogonally *to the orientation of the street on which the corresponding address lies. This is in sharp contrast to the *coincident *alignment of automated geocoding errors with the axial orientation of the street, which we noted previously (Figure 2).

The orthogonal alignment of E911 errors occurs as a result of offset errors of substantial magnitude, which in turn are due to the definition of the E911 geocode in rural areas as the coordinates of the intersection of the public road and private road leading to the residence, coupled with the approximate perpendicularity (in most cases) of the angle between the public and private road. The outliers, for the most part, correspond to those cases for which the offset is relatively large and the private road meanders in such a way that a hypothetical line segment connecting the residence to the public road-private road intersection is far from being perpendicular.

**Figure 5:**
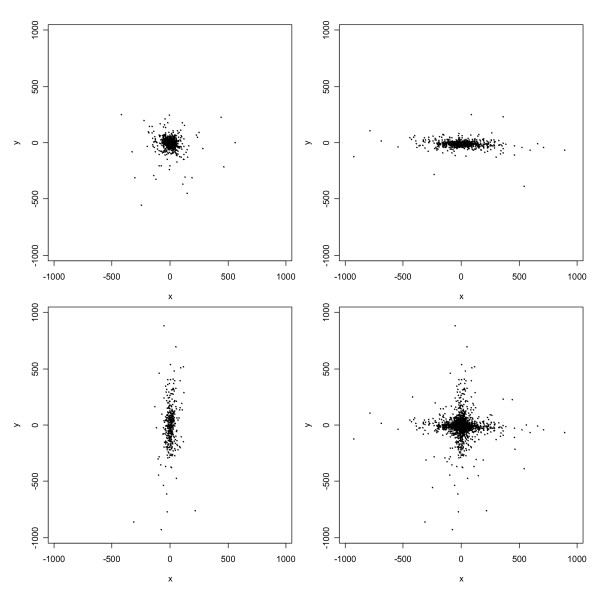
Simulated data from the fitted three-component t mixture distribution for the E911 geocoding errors. The upper left panel, upper right panel, and lower left panel correspond to components in order of decreasing *p*_*i*_; and the lower right panel is their superposition.

Normal and t mixture distributions with various numbers of components were fitted to the E911 errors. Values of *BIC *for these fits are listed in Table 1c. On the basis of these values, it appears that a three-component t mixture model provides the best fit; normal models, as well as t models with less than three components, are inadequate. Likelihood-based parameter estimates for the three-component model are given in Table 2c in order of decreasing *p*_*i*_, and Figure 5 displays 2354 simulated observations from the fitted model. Note that the means of all components lie fairly close to the origin, indicating little systematic bias in the errors. The estimates and component plots reveal that the component comprising the largest proportion (about 52%) consists mostly of relatively small (standard deviation just over 60 m), nearly isotropic errors. The other two components (comprising about 29% and 19% of the errors, respectively) correspond to errors tending to be of larger size (standard deviations of 290 m and 354 m) lying close to the E-W and N-S axial directions, respectively. All three components are quite heavy-tailed, thus outliers occur in all of them. Overall, the simulated data (Figure 5, lower right panel) again seem to reproduce the observed data (Figure 4, upper left panel) quite well.

The lower right panel of Figure 4, which displays all of the E911 errors relative to the axial orientation of the corresponding street segment, highlights the aforementioned orthogonality of the errors to street orientation. Normal and t mixtures, once again, were fitted to the errors in this plot. Values of *BIC *and likelihood-based parameter estimates are given in Tables 1d and 2d, respectively. According to these results, a two-component t mixture is best-fitting. The component comprising the largest proportion (70%) consists of relatively small errors that are, on average, about twice as large in the orthogonal direction as in the coincident direction. The remaining component consists of much larger errors that average about seven times larger in the orthogonal direction than in the coincident direction. Both components are rather heavy-tailed, indicating that outliers occur regularly for both.

## Conclusion

The major question motivating this investigation was whether one could find useful models for the probability distribution of positional errors associated with geocoding, i.e. models that are sufficiently rich to adequately fit various geocoding error datasets yet sufficiently parsimonious to be practical for use as measurement-error models for statistical analysis. The answer to this question, based on our findings, is solidly (though not unequivocally) in the affirmative; and the class of models that seems best suited for the purpose is the class of mixture models of bivariate t distributions. These models can adequately fit such features as clustering along several axial directions, systematic bias in any direction(s), and outliers, all of which occurred in our data; simpler models such as uniform and normal distributions, which have been used previously for positional errors in spatial data, cannot. Moreover, t mixture models are feasible for use with emerging applications of measurement-error methodology to epidemiologic research [19,22], provided that they consist of very few components. Based on our results and the other published graphical displays of geocoding errors of which we are aware [12,14], we conjecture that a mixture of three (two) t distributions will usually be sufficient for errors (aligned errors) associated with 100%-matched automated geocoding and E911 geocoding, but additional investigations in other places are needed to substantiate this. Positional errors from regions with less rectilinear road networks than Carroll County may not require as many components, as they are less likely to exhibit clustering in the E-W and N-S axial directions; a case in point is displayed in [14]. In some cases a single t distribution or, in the unlikely event of no outliers, a single normal distribution may even suffice. In any case, if the analyst assumes a t mixture model either with more components than necessary or when a normal mixture model will suffice, the *BIC*-based model selection procedure we have described will (with high probability) point the way to the simpler model.

The one situation we encountered in which mixture models of t distributions proved to be less than fully successful occurred with automated geocoding errors for which an address-matching threshold of less than 100% was used. In this situation, a few small clusters of extremely large errors occurred. Such errors are difficult to model parsimoniously and, regardless of how they are modeled, will weaken the conclusions made from subsequent statistical inferences using measurement-error methodology. Consequently, we recommend using only 100%-matched addresses for spatial epidemiologic analyses.

Our investigation indicated that t mixture models were equally useful for 100%-matched automated geocoding errors and E911 geocoding errors, despite some differences in their distinctive features. In particular, t mixtures were able to accommodate the difference in the major axis of error alignment relative to the alignment of the corresponding street (parallel for automated geocoding, perpendicular for E911 geocoding). The error distributions associated with other geocoding methods may have their own distinctive features (see [14], for example, for a graphical display of errors incurred by parcel address-matching), and it remains to be seen whether t mixtures are as successful for them.

Further investigation is currently underway to determine if t mixture models are as useful for positional errors corresponding to non-rural addresses as they appear to be for rural address positional errors and, if so, how the components might differ from those for rural addresses. Results from previous studies of positional errors for datasets combining both rural and non-rural addresses [38,10,11,14] suggest strongly that component variances will be smaller for non-rural addresses, but we refrain from predicting how many components may be needed and whether they will prove to be heavy-tailed, mean-shifted away from the origin, etc. Future research may also address the modeling of the probability distribution of positional errors associated with reverse address-matching [39].

How might the methods developed here be adapted to the common situation in which it is not possible to obtain a "gold standard" geocode for each address that has been geocoded via automated geocoding? In some cases it may be feasible to obtain the more accurate geocode for a randomly selected portion of the addresses, from which the probability distribution of positional errors associated with automated geocoding may be estimated. This estimated distribution may then, as a practical matter, be presumed to apply to the entire set of addresses. In those cases where no sample of positional errors can be obtained, it may still be possible to estimate parameters of a probability distribution of positional errors, provided that a parsimonious model for the true locations of addresses is known (up to its unknown parameters). An illustration of this can be found in [22], and others will be reported elsewhere.

In focusing our attention on geocoding errors, we have ignored the fact that for many studies, automated geocoding is incomplete; that is, not all addresses can be assigned point-level spatial coordinates by the software. In fact, it is common in practice for 20% or even as many as 40% of subjects' addresses to fail to geocode using standard software and street files. For example, Gregorio et al. [40] and Oliver et al. [41] present public health studies in which 14% and 26%, respectively, of addresses could not be assigned a point location via automated geocoding, and for our exclusively rural address dataset this figure was even higher (38%). A statistical analysis based on only the observations that geocode is subject to selection bias [42,41]. However, there is virtually always a reliable coarse (areal-level) measurement, e.g. a zip code, associated with each observation that fails to geocode. Coarse locational data may be combined with the observed point-level data to make valid statistical inferences in the presence of geographic bias via either (a) a coarsened-data maximum likelihood estimation procedure [43], or (b) imputation of a surrogate point location (such as that of a randomly selected event within the same zip code) for the addresses that do not geocode [44]. Fully satisfactory inference procedures for data whose point locations are ascertained by automated geocoding may require that an inference procedure developed for use with incompletely geocoded data be combined with modifications to account for positional errors.

## Authors' contributions

DLZ conceived of this study and drafted the majority of the manuscript. DLZ also directed, and XF performed, the statistical analysis. SM performed the automated geocoding and oversaw the orthophoto geocoding of the Carroll County data and contributed to the writing of the Methods section. GR contributed to the writing of several sections.
